# An Editorial Anabasis

**Published:** 1910-12

**Authors:** 


### EDITORIAL DEPARTMENT.

### AN EDITORIAL ANABASIS.

I am going to get off of my base. But, unlike Cyrus and Xenophon, who left theirs on a memorable occasion, I'm not "going up from the coast," nor "into the heart of Asia," but I'm going to make a flight to the stars; break Ralph Johnstone's record for altitude,—make an excursion into the green alfalfa fields of fancy and roam into the stubble pastures once green but now brown and sear—the past—yet which furnish still fairly good gleaning. For,—I take it, doctors, like horses, get tired of an eleven months' diet of dry stuff,—the doctors, not the horses, are fed on fever, pus and bile; on interesting cases, clinical reports, alleged editorials, book notices and reading notices every month, *ad nauseam,* and I believe they, like the horses, will relish a little green stuff. So—here goes.

**Itur ad Astra.**

Did you ever reflect upon the exquisite and eloquent beauty of human environment, the grand pyrotechnics of the moonless night sky—where,

"Silently, one by one, in the infinite meadows of heaven blossom the lovely stars, the forget-me-nots of the angels?"

And—realize that every "star" is a sun, the center of a system of worlds—planets—like the little eight-thousand-mile ball we live upon, and which, to ninety-nine hundredths of the human family, is "the world?" That there are 140,000,000 of those suns in the Milky Way alone, and that our nearest neighbor—Alpha, in the Centaur—is so far away that a ray of light emitted from that star traveling 186,000 miles a second, or about eight times around the earth, will require four and a half years to reach us, and that, should Alpha be suddenly blotted out, we would continue to see it in the year 1915? Did you ever know that the Milky Way is the work-shop of worlds, where sun-building is going on all the while,—and that with a good glass you can see,—in Orion, the great spiral nebula being shaped into a sun, which, in perhaps a million years or less, will burst upon the dwellers on earth as a star of the first magnitude—another diamond in the sword of the Wild Huntsman, who is still chasing the daughters of Atlas and Pleione round and round—preceded by his big dog Sirius and followed by his little dog Procyon? Did you ever watch the rising of the well known constellations in the Zodiac—see the fiery Scorpion—the boogaboo that frightened the horses hitched to the chariot of the sun—that time when old Phoebus indiscreetly let his boy Phaethon drive over the course, and made them run away? and see the Archer in the act of firing an arrow into the business-end of old Scorpio? It is a lovely night sight all summer, when the moon is engaged elsewhere. Did you ever watch the Lion come up from his lair—spitting fire—(the Leonids) giving us a grand pyrotechnic display—in August and November each year, and a grand matinee performance once every thirty-three years? No. I bet you didn't. Nine-tenths of mankind live their little narrow lives and perish like the leaves, without ever looking above; without seeing the constant moving panorama of "the heavens that declare the glory of God and show forth his handywork"; or,—if they do,—they have eyes that see not, or—seeing—they know not what it is, nor what it means. They are too busy making money; for they say:

Lives of rich men all remind us
That we must make our little pile;
And departing leave behind us,
Cash. Our wives must dress in style.

Do you see any grandeur in a storm? realize that the winds and the waves, the suns and the stars, the blossoms and the birds and the bees, the lightnings and the thunder, are all manifestations of the great "I AM," whose glory, goodness, might and power are all the while displayed for our enlightenment and enjoyment of His goodness, love and abundant providence?

I love the stars. My wife was out of town all summer, and I had to "associate with myself," as Dick Ledbetter (dear old fellow; he's over there long ago) would say. I didn't get lonesome; I'm one of those fellows who find resources in themselves. I realize Bryant, who says:

"To him who in the love of nature holds communion with her visible forms she speaks a varied language."

I love to listen to that kind of talk. I love to hold communion with her visible forms.

One sultry night in August, I sat alone in the park—holding said communion—gazing into the depths of the glorious star-lit sky,—when a fellow came and sat down on the bench by me;—I take it that he was a Rube, or he may have been a member of the

Legislature, then in session—many of them were Rubes—or Hill Billies—and he said:

"Lonesome?"

I said: "No, I'm not lonesome." (But I was chock full of talk and would have been glad of a chance to fire it off. I believe I would have talked to a cat just then.) I said: "I am communing with nature. She is eloquent tonight." "For one's gayer hours she has a voice of gladness, and a smile, and eloquence of beauty, and she glides into his darker musings with a mild and healing sympathy that steals away their sharpness, ere he is aware." See! "The stars that oversprinkle all the heavens seem to twinkle with a crystalline delight, keeping time, time, time, with a sort of runic rhyme." "Do you love the stars?"

"Yes," said he, "I knew a pretty little poem about them once." "Twinkle, twinkle, little star, how I wonder what you are."

I ran all the way home, and I've been wondering ever since whether that darned fellow wasn't guying me, and which of us was the fool.

Now for the stubblefield, gleaned over, reaped years ago. Roaming over it lately, I stumbled upon a small sheaf that had been gleaned ("Red Back," October, 1900), and as I have a great many subscribers that have joined the procession since then, I reproduce it here. It is one of my most pleasant memories. It goes something like this:

Down on the Farm.—"Give us a rest, oh, give us a rest," I sighed. Well, we've had a rest, and during the time have done some hard work. We haven't exactly experienced James Whitcomb Riley's "Knee-deep in June," but have lazed during the sweltering days of August, lolling on grassy lawns, beneath breezy trees-es, and have read all the late novels (that was the hard work). In the cool of the afternoon with the meek and lowly earth worm we'd angle for the frolicsome mudcat, or beguile the festive perch in the turbid waters of the Colorado close by the ranch where we ranched; have watched the sun set, reveled in the delights of sixty-pound watermelons, and eaten of yaller-leg-pullets (fryin' size), great store. We plucked the luscious "Chinese-clings" and other sun-kissed peachy-cheeked clingers, to say nothing of cantaloupes. We have strolled down the meadow,—late of evenings—and watched the whistling mower as he moved the Johnson-grass, and tumbled on the mounds of new-mown hay, like a schoolboy, repeating, the meantime, those beautiful lines of Alfred Austin's: "Maud Muller's brother Jake raked the meadow with a two-mule rake." Oh, it's great. It makes a fellow poetic. Afternoons we would stroll down to the river bridge and see the picture Mrs. Townsend so beautifully describes of a summer day in the country:

"The soft wandering gale fills a silvery sail
That idly floats by on yon far-away stream;
A frail spirit-boat 'neath the other doth float,
Faintly fair, like some beautiful dream of a dream."

It was a big thing, too, to see

"The bee from the bosom of the red clover blossom,
As he hurried to sip of the buckwheat in bloom;
While the down of the thistle and the blackbird's clear whistle,
Were blent with the summer day's light and perfume."

But the swelling on our editorial chin was bigger, where one of those same bumble bees got in his work once, while I was trespassing on his preserves—his favorite figs (fig preserves), which he and his were putting up expressly for family use.

Much has been said and sung of the charms of pastoral life, and the milkmaid in her dainty cap has been an idyl,—an ideal which even queens have delighted to personate. Marie Antoinette didn't know a thing about milkmaids; she ought to have seen ours, out on King's Ranch. In this instance "she" was a big fat fellow with a sun-hurter straw hat and one gallus. To see him milk was not an idyl; nor was it an idle speculation, but more than a suspicion, that the milk was sterilized or fertilized by great drops of perspiration which gathered on the old fellow's brow about the size of a biscuit. Mrs. Townsend sang of

"Brownie and Daisy, milk-laden and lazy,
And the gentle-eyed heifer half standing aloof,
While the dew-dimpled grass softly yields as they pass
To the lingering print of each slowly-raised hoof."

That's real pretty,—isn't it? Our Brownie was a white cow,—a muley, and she was a kicker from away back. When she'd kick the milkmaid with the gallus and straw hat he'd kick her. There was an alleged wag on the ranch, who was of a poetic turn. With his corn-cob pipe in his mouth, he'd saunter idly about, wishing something would happen; and when it did happen,—when the big aerolite passed over one night, he dodged and ran under the cow-shed. He'd say:

"Doctor, come, come into the deep-tangled wildwood, and let us see the cornucopias cope and the honey-suckles suck."

Not much I would; too much exertion. But I stopped to bandy words with him, for which I despise myself. It were pleasanter, I told him, to loll on the broad verandas in a hammock, and listen to the lilacs lie; or stroll into the meadow, and in the gloaming listen to the cornstalk, and hear the May-pops pop; while the dear little grasshoppers sang, "In this Wheat Bye and Bye."

Alas, that all things fair and bright must fade. Summer has gone, and already the bumble bees are humming of autumn days coming. Soon the frost will be upon the pumpkins and they will cease to pump. Back, now, to the harness and the mill, to grind out the despised but indispensable and ever elusive dollar, which no family can afford to be without.

Heigh-ho! Jolly—and the crowd jollies with you. Grouch and you grouch alone. One smile is worth a dozen sighs. Selah!

